# Bioselective PES Membranes Based on Chitosan Functionalization and Virus-Imprinted NanoMIPs for Highly Efficient Separation of Human Pathogenic Viruses from Water

**DOI:** 10.3390/membranes12111117

**Published:** 2022-11-09

**Authors:** Carmen Andreina Olivares Moreno, Zeynep Altintas

**Affiliations:** 1 Institute of Chemistry, Faculty of Maths and Natural Sciences, Technical University of Berlin, Straße des 17. Juni 124, 10623 Berlin, Germany; 2 Institute of Materials Science, Faculty of Engineering, Kiel University, 24143 Kiel, Germany

**Keywords:** polymeric membrane, membrane hydrophilic modification, chitosan as spacer arm, bioselective membrane, virus-imprinted polymers, molecularly imprinted nanoparticles

## Abstract

Waterborne viruses are a public health concern due to relatively small infection doses. Particularly, adenoviruses (AdVs) are more resistant than RNA viruses to water purification treatments in terms of ultraviolet (UV) irradiation, pH, and chlorination tolerance. Moreover, AdVs are one of the most predominant waterborne viruses. Membrane separations have proven superior removal capabilities of waterborne pathogens over other separation methods. However, virus removal at ultratrace levels is still a significant challenge for current membrane technology. This study successfully addressed this challenge by developing a bioselective polyethersulfone (PES) membrane by a joint strategy involving chitosan hydrophilic surface modification and the immobilization of adenovirus-specific molecularly imprinted nanoparticles (nanoMIPs). The topological and chemical changes taking place on the membrane surface were characterized by using atomic force microscopy (AFM) and scanning electron microscopy (SEM). Furthermore, hydrophilicity and membrane performance were investigated in terms of swelling behavior, permeation flux, and surface fouling studies. The membrane efficacy was evaluated by filtration experiments, where the virus concentration of the loading solution before filtration and the permeates after filtration was quantified. The novel bioselective membrane showed excellent virus removal capabilities by separating 99.99% of the viruses from the water samples.

## 1. Introduction

Clean water access has been essential for the development of human society. However, numerous waterborne diseases affect millions of people worldwide as a consequence of contaminated water consumption [1]. Over 400 waterborne pathogens have been identified including viruses, bacteria, and parasites [2]. Viruses are particularly difficult to remove from water sources due to their small size, high mobility, resistance to chlorination and lower temperatures, and their capability to survive within wider pH ranges (3–10) [3,4,5]. In addition, viruses can cause infections even from relatively low dose concentrations. The risk of infection after consuming water contaminated with viruses is 10 to 10,000 times greater than pathogenic bacteria with similar concentrations [4,6]. Previous works have reported that up to 10^11^ waterborne viruses per gram of feces from infected individuals have been detected and the main infection route is fecal–oral [7].

Adenoviruses (AdVs) are one of the most predominant waterborne viruses. For example, 87.5% of the investigated water samples of an enteric virus assessment performed in the urban river Ruhr (Germany) were positive for human AdVs [8]. Adenoviruses are associated with a broad range of symptomatic infections in the respiratory system, eyes, gastrointestinal tract, central nervous system, and genitalia [4]. Additionally, adenovirus transmission is not limited to the fecal–oral route. AdVs are alternatively transmitted via oral–oral, hand–eye, and via inhalation of virus particles in an aerosol route [9]. 

Water treatment has played a crucial role for contaminant and pathogen removal from water sources, especially from sewage. Generally, water treatment involves primary, secondary, and tertiary treatments. Secondary sewage treatments are not efficient enough to remove pathogens from water streams as previous works have reported [10]. Consequently, tertiary treatments are fundamental for removing pathogens from wastewater and ensuring a clean water supply.

Tertiary water treatments include ultraviolet (UV) irradiation, chlorination, and membrane filtrations [10]. The first two methods are commonly used in water treatment plants. Nevertheless, AdVs are resistant to chlorination and approximately 60 times more resistant to UV irradiation than RNA viruses. The damaged portion of the double-stranded DNA of AdVs after UV irradiation could be repaired by the host enzymes using the undamaged DNA-strand portion as template [4]. 

Membrane technology has been proven to reduce the concentration of viral pathogens in a more efficient way in comparison with other water treatment methods [11]. Moreover, membranes are fabricated in a wide range of materials including ceramics and polymers [12]. Particularly, polyethersulfone (PES) membranes have several advantages including chemical resistance, a high glass transition temperature (above 220 °C), a relatively low cost, non-toxicity, and mechanical stability [13,14]. 

The principal membrane separation methods of pathogenic viruses from water include reverse osmosis (RO), microfiltration (MF), ultrafiltration (UF), and nanofiltration (NF) [15]. Membrane separations have additional advantages, e.g., ease of operation and relatively small environmental footprint [16]. Nevertheless, the virus separation efficiency of commercial membranes is limited. Previous works have reported that conventional UF membranes are able to remove up to 90% virus concentration [12] meaning that a minimum 10% virus concentration remains in water after filtration. Additionally, surface fouling and lack of specificity as well as selectivity towards viral pathogens, especially at trace levels, are still significant challenges for membrane separations [7,10,17].

The use of recognition elements specifically targeting waterborne adenoviruses represents a promising opportunity for selective separation of these pathogens from water. These receptors include antibodies, aptamers, and molecularly imprinted polymers (MIPs) [18]. During past decades, research works have been oriented to developing artificial recognition elements (e.g., MIPs) that are more stable and cost-efficient than biological antibodies. Particularly, MIPs are compounds with tailor-made binding sites that present complementary shapes, sizes, and functional groups of their template molecules, e.g., whole viruses [19,20]. 

The format of the MIPs depends on the synthesis method. For example, MIP nanoparticles (nanoMIPs) prepared by solid-phase synthesis are highly suitable for covalent immobilization on the flat sensor surfaces for achieving sensitive and specific detection of viruses [21,22]. Recent works have highlighted the great potential of virus-imprinted nanoMIPs as nanomaterial for the identification, characterization, and separation of viruses for diagnostics, point-of-care detection, and separation technologies [22,23]. 

Molecularly imprinted membranes (MIMs) are special membranes with recognition sites that specifically bind to a particular target [19]. MIM technology efficiently combines the separation capabilities of membranes with the selective recognition capabilities of MIPs. MIMs enable a wide range of new possibilities to detect and remove waterborne pollutants including endocrine disruptors and pathogenic viruses [19,24]. Nevertheless, the steric hindrance created by MIP particles on the surface limits the selective binding with the target. Previous studies have demonstrated that the insertion of molecular linkers to create a distance between the surface and the recognition elements favored specific binding, especially for relatively large receptors [25] such as virus-imprinted MIPs. 

Chitosan (Chi) is a hydrophilic, non-toxic, biodegradable, antibacterial, and biocompatible natural polysaccharide [17] that plays a key role in the enhancement of the hydrophilicity of membranes. In this study, commercial PES membranes were functionalized by chitosan hydrophilic modification for the subsequent immobilization of nanoMIPs for the first time. The chitosan molecules created spacer arms between the membrane surface and the receptors that enhanced the accessibility of the immobilized nanoMIP binding sites on the surface, thus favoring the specific binding of adenoviruses to nanoMIPs. Our innovative immobilization strategy has resulted in a highly efficient removal of adenoviruses from water samples by providing a bioselective filtration system that brings the beneficial features of chitosan, imprinted polymers, and membrane technology together.

## 2. Materials and Methods

### 2.1. Chemicals and Reagents

Commercial PES membranes (Supor^®^-PES membranes) were purchased from Pall Corporation in 50 mm diameter membrane disk format with a pore size of 0.45 μm. Low molecular weight chitosan (deacetylated chitin, poly(D-glucosamine)) was supplied by Sigma-Aldrich Co., (St. Louis, MO, USA) molecular weight 50,000–190,000 g mol^−1^. ReagentPlus^®^ glacial acetic acid was provided by Sigma-Aldrich Co., (concentration ≥ 99%). Sodium hydroxide (NaOH) in pellet format was supplied by VWR Chemicals BDH Prolabo, ≥99%, molecular weight 40 g mol^−1^. Glutaraldehyde (GA) 25% solution in water was provided by Merck KGaA, and ethanol absolute 99.9% was supplied by VWR Chemicals BDH Prolabo. Hydrochloric acid (HCl), 37% concentration, molecular weight 36.46 g mol^−1^ as well as acetonitrile (ACN), HPLC grade, ≥99.9%, molecular weight 41.05 g mol^−1^ were provided by Sigma-Aldrich Co., Finally, phosphate buffered saline (PBS) was purchased from Sigma-Aldrich Co., in tablet format and inactivated adenovirus serotype 5 was provided by Free University of Berlin.

### 2.2. Membrane Functionalization with Chitosan

Chitosan solution (1.0 wt% chitosan) in diluted acetic acid (2.0 wt% acetic acid in double-distilled water) was freshly prepared for every membrane preparation day. After adding the chitosan powder to the diluted acetic acid, the solution was vortexed and ultrasonicated for 15 min to enhance homogeneity.

Untreated bare membrane samples were cut from commercial PES membrane disks into smaller pieces of 3 × 3 cm. After that, the samples were wetted by filtering double-distilled water for five minutes and were then immersed in chitosan solution for 120 s. Thereafter, the membranes were dried by annealing at 60 °C for 45 min under vacuum conditions.

The annealing step was followed by neutralization with diluted NaOH solution. Therefore, the dried membrane samples were immersed for 30 min in 1.0 M NaOH solution in a 50 vol% water–ethanol mixture. After neutralization, the samples were washed with 50 vol% ethanol in double-distilled water for 10 min followed by washing with pure double-distilled water for 30 min. The samples were then dried for one hour in the desiccator under vacuum conditions.

### 2.3. NanoMIP Immobilization

**Figure 1:**
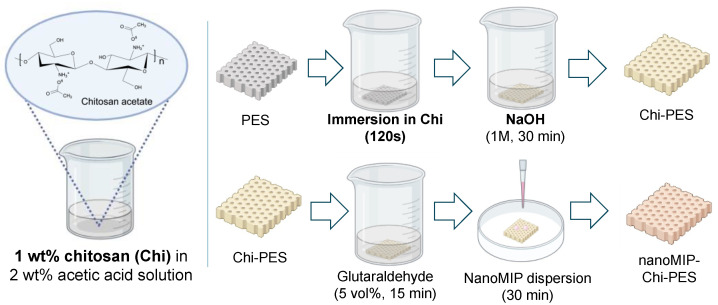
Schematic representation of the main modification steps of polyethersulfone (PES) membrane with chitosan (Chi) and the immobilization of molecularly imprinted nanoparticles (nanoMIPs).

Adenovirus-imprinted nanoMIPs were synthesized by following a previously reported solid-phase synthesis method involving template immobilization on glass beads, polymerization of functional monomers and crosslinkers, as well as elution of nanoMIPs after a cold (15 °C) and hot wash (60 °C) with double-distilled water [21]. After nanoMIP preparation, a dispersion of nanoMIPs in double-distilled water was prepared. The resulting nanoMIP dispersion with a concentration of 500 µg mL^−1^ was ultrasonicated for five minutes, aliquoted in 400 µL vials, and stored in the freezer at −20 °C. Chitosan functionalized membrane samples were immersed in an aqueous glutaraldehyde solution (5 vol%) for 15 min. Thereafter, 200 µL of previously vortexed nanoMIP dispersion were deposited on top of the membrane surface with a micropipette. After 30 min, membranes were dried in the desiccator under vacuum for one hour. Figure 1 shows a visual representation of the main modification steps of the PES membrane with chitosan and nanoMIP immobilization.

### 2.4. Characterization of Membranes

#### 2.4.1. Contact Angle Measurements and Swelling Behavior

Contact angle measurements were performed in the DataPhysics optical contact angle meter OCA 20 by the pending drop method. The water contact angle was measured in five different areas of the membrane by depositing a droplet of water on the surface. The output data were analyzed with the software SCA 20 provided by DataPhysics.

#### 2.4.2. AFM Characterization

The membrane surface was characterized by atomic force microscopy (AFM). Particularly, surface topography, phase distribution, and root mean square (RMS) roughness were measured by AFM NanoWizard II (JPK Instruments AG., Germany). Image data were processed using the JPK Data Processing software. AFM height and phase micrographs were scanned and analyzed for each sample in areas of 50 × 50 μm, 10 × 10 μm, and 3 × 3 μm. The measurements were done in intermittent contact mode in air at room temperature and the samples were placed over a glass plate. Regarding the feedback parameters, the IGain was set between 30 Hz and 60 Hz, with 50 Hz being the preferred value for most samples with a larger scanning; the PGain was between 0.002 and 0.004, with 0.002 being the preferred value for most samples. Furthermore, for the image settings, the line rate was set to 0.2 Hz for all samples to ensure high-resolution micrographs. The resulting image data were analyzed using the JPKSPM Data Processing software. 

#### 2.4.3. SEM Imaging

The membrane surface was also studied by employing a scanning electron microscope (SEM, a GeminiSEM 500, Zeiss, Germany) with an accelerating voltage of 6 kV, magnifications of 10,000× and 100,000×, and an InLens detector. Images were taken with a drift compensation frame average with an aperture size of 7 μm in a high vacuum mode. Before the SEM measurements, gold was sputtered on the surface to enhance contrast by using a gold sputtering machine Balzers Union FL 9496 SCD 030 with 5 mA of power of electrical field. For this, the argon plasma and vacuum were first set to 0.1 mbar. Moreover, the gold layer thickness was set to 4 nm.

### 2.5. Membrane Performance Experiments

**Figure 2:**
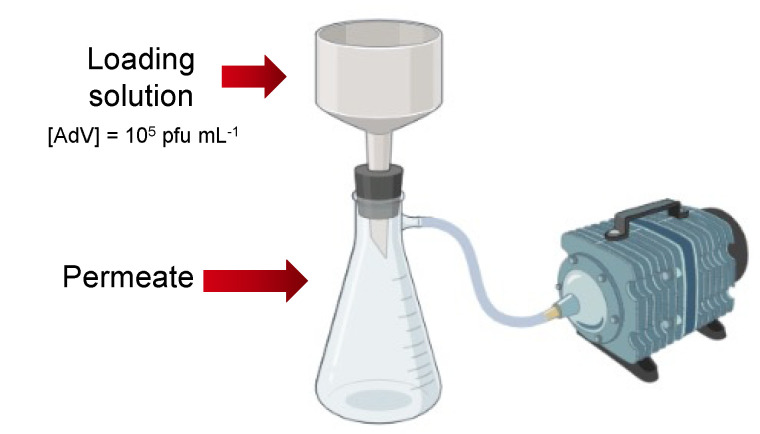
Schematic representation of filtration experiments.

Membrane performance was evaluated by filtering different volumes of water solution containing a known concentration of adenovirus. The filtration setup is shown in Figure 2. Loading solution with a known concentration of inactivated adenovirus (10^5^ pfu mL^−1^ where pfu stands for plaque-forming unit) was poured into a 10 mL Büchner funnel with a membrane sample. This funnel was on top of a filtration flask connected to a vacuum pump (pressure 0.03 mbar) that collected the permeate or filtrate, which is the solution after going through the membrane. 

A new membrane was used for every filtration experiment, and these membranes had an average diameter of 2.5 cm. Two different loading solution volumes (5 mL and 10 mL) were evaluated for the bare PES membrane, chitosan functionalized membrane, and the chitosan functionalized membrane with immobilized nanoMIPs.

The virus concentration of the permeates resulting from the bare polymer membrane and the bioselective membranes with nanoMIPs filtrations was quantified by a quartz crystal microbalance (QCM) analysis. Firstly, a calibration curve was achieved with a concentration range between 10 and 10^7^ pfu mL^−1^. Then, the permeates were analyzed immediately after filtration of the loading solution. A different QCM chip was used for every loading solution volume. 

The loading solution was prepared in PBS in Millipore water previously filtered with a syringe filter (0.20 µm pore size). To start the measurement, degassed and filtered PBS was injected into the QCM measurement chamber (volume: 200 µL). Then, a solution with 500 µL 1-ethyl-3-(3-dimethylaminopropyl)carbodiimide (EDC, 0.4 M) and 500 µL N-hydroxysuccinimide (NHS, 0.1 M) was injected over a duration of four minutes. After that, PBS was injected again and when reaching an optimal temperature and stable frequency response, the permeate from the filtration through the bioselective membrane was injected, followed by the permeate from the filtration with a bare polymer membrane and the loading solution. Between every injection, the surface was briefly rinsed with PBS.

Additionally, the regeneration capabilities of the membranes were evaluated by using the filtration setup shown in Figure 2. First, 10 mL loading solution was filtered through the membrane. Then, 10 mL regeneration medium was filtered through the same membrane. After that, 10 mL PBS was filtered, followed by a second filtration of 10 mL regeneration medium and then 10 mL PBS through the same membrane. Two distinct regeneration media were investigated: diluted HCl (0.1 M, room temperature) and ACN (first filtration: 15 °C, second filtration: 60 °C). The permeates (from the loading solution and PBS filtrations) were analyzed by QCM measurements.

## 3. Results and Discussion

### 3.1. Contact Angle Measurements and Swelling Behavior

The commercial bare PES membranes used in this work were already hydrophilic and swelled when water droplets were placed on the surface. This behavior was observed by contact angle measurements. Previous studies have described the swelling behavior of PES membranes. It can be quantified by Equation (1), where *Q* is the degree of swelling, *ρ_s_* is the density of the solvent, *W_dry_* is the weight before adding the solvent on the surface and *W_wet_* is the weight after adding the solvent [26].

(1) $$\;\;Q=\frac{1}{\rho_{s}}\frac{W_{wet}-W_{dry}}{W_{wet}}$$

Table 1 shows the weights of the membrane samples before and after adding 1 mL water to the membrane surface. The degree of swelling was calculated by using Equation (1). The investigated membrane samples exhibited significant swelling behavior; even the bare PES membrane absorbed water deposited on the surface. Previous works have reported a degree of swelling of 1.33 for PES membranes [26], which is comparable to the experimental values (1.08–1.26) shown in Table 1. Furthermore, the degree of swelling increased after every major treatment step. This trend might be indicative of an increase in the hydrophilicity of the membrane samples.

**Figure 3:**
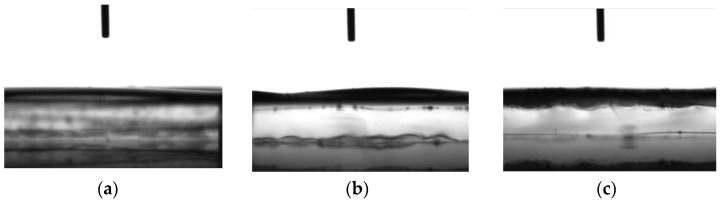
Goniometer pictures of (**a**) bare PES membrane, (**b**) membrane before nanoMIP immobilization, and (**c**) membrane after nanoMIP immobilization.

Furthermore, the height of the cross section of the membrane surface changed considerably after water droplet deposition. During a contact angle measurement, a video of the solvent droplet deposition was recorded by the goniometer. Figure 3 shows pictures from the water contact angle video corresponding to different steps of membrane functionalization. In all the studied samples, the cross section has changed after the water droplet deposition. 

The water droplet was absorbed immediately after deposition on the membrane surface, before any contact angle was observable, and then further processed by the goniometer software to generate the corresponding data files. The chitosan functionalization plays a less prominent role in enhancing the hydrophilicity of membranes that are already hydrophilic before modification compared with hydrophobic untreated membranes undergoing chitosan modification [16]. Even though the swelling behavior of PES membranes hindered the contact angle measurements, it was possible to investigate the hydrophilic characteristic of the membranes before and after nanoMIP immobilization. Given the degrees of swelling displayed in Table 1, the investigated PES membranes were hydrophilic before and after performing the treatment steps.

### 3.2. AFM Characterization 

**Figure 4:**
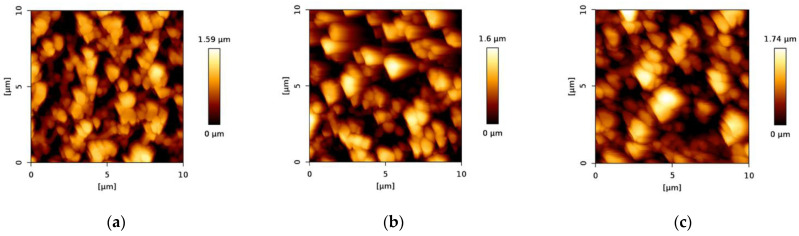
AFM micrographs of two-dimensional height images in 10 × 10 µm scale of (**a**) bare PES membrane, (**b**) chitosan functionalized PES membrane before nanoMIP immobilization and (**c**) chitosan functionalized PES membrane after nanoMIP immobilization.

**Figure 5:**
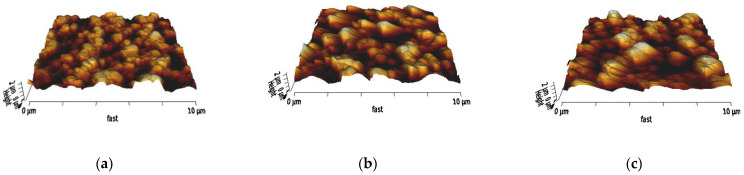
AFM micrographs of three-dimensional topology in 10 × 10 µm scale of (**a**) bare PES membrane, (**b**) chitosan functionalized PES membrane before nanoMIP immobilization, and (**c**) chitosan functionalized PES membrane after nanoMIP immobilization.

**Figure 6:**
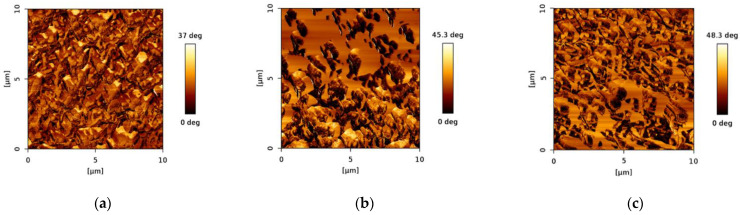
AFM phase images of (**a**) bare PES membrane, (**b**) chitosan functionalized PES membrane before nanoMIP immobilization, and (**c**) chitosan functionalized PES membrane after nanoMIP immobilization.

The surface of the membrane samples was characterized by AFM, where three different scanning areas, including 50 × 50 μm, 10 × 10 μm, and 3 × 3 μm, were investigated. Moreover, the 2D and 3D topology, as well as the phase image, were studied before and after chitosan and nanoMIP immobilization (Figure 4, Figure 5 and Figure 6). In addition, the root mean square roughness (RMS, also known as Rq) was determined in different areas of each 2D topology micrograph and approximately 10 RMS measurements were taken per sample.

The functionalization steps increased the roughness. This trend can be particularly observed in the height images (Figure 4), where the scale bars beside the micrographs show an increasing maximum value of the height. Firstly, the maximum height slightly increased from 1.59 µm (Figure 4a, bare PES membrane) to 1.60 µm (Figure 4b) after chitosan functionalization. This maximum height further increased to 1.74 µm (Figure 4c) after nanoMIP immobilization.

This trend was also observed in the RMS values, and it was consistent among all the scanning areas (Table 2). After chitosan functionalization, the roughness increased and after nanoMIP immobilization, the RMS roughness further increased. 

Furthermore, phase images show a similar trend in the maximum degree (Figure 6). In other words, the phase degree increases after major functionalization steps. The phase distribution is quite uniform in the bare PES membrane (Figure 6a) since the chemical composition of such a membrane is only PES. However, when functionalizing the membrane with chitosan, the chemical mapping looks significantly different as displayed in Figure 6b. There are prominent contrasts in the phase distribution pattern indicating different chemical phases, which is expected after functionalization with another chemical (chitosan, which was neutralized by treatment with NaOH). Moreover, the distribution pattern is more irregular than in the bare PES membrane.

Upon nanoMIP immobilization, the chemical phase distribution pattern became more regular and the chemical phases were more homogeneously distributed on the surface as can be observed in Figure 6c.

### 3.3. SEM Imaging of PES Membranes

**Figure 7:**
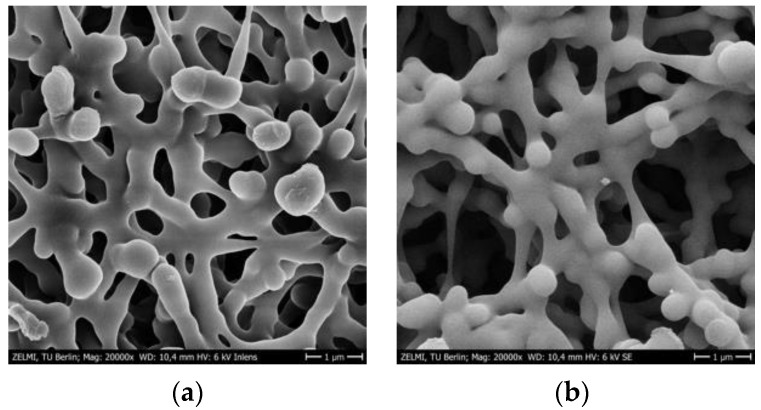
SEM micrographs of bare PES membranes recorded with (**a**) the InLens detector and (**b**) the secondary electron detector (SE).

The membrane samples were further studied by SEM in order to better understand the surface morphology after major steps of membrane preparation. A gold layer was sputtered on the surface to improve contrast because many polymers such as PES do not conduct electricity. In fact, the resolution of the SEM micrograph is significantly poor without the gold layer. Moreover, the electron beam detector of the scanning electron microscope is another important consideration for the SEM studies. Figure 7a,b show a comparison between the micrographs resulting from using two different detectors located at two distinct positions of the microscope. 

**Figure 8:**
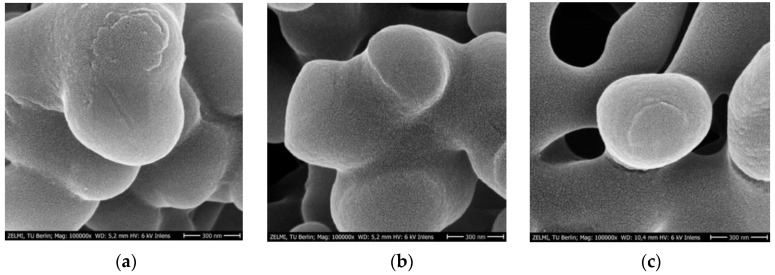
SEM micrographs at 100,000× magnification of (**a**) bare PES membrane, (**b**) chitosan functionalized PES membrane before nanoMIP immobilization, and (**c**) chitosan functionalized PES membrane after nanoMIP immobilization.

The SEM image measured with the InLens detector (Figure 7a) located in the electron beam path exhibits excellent contrast for the membrane structure and even the cracks from the gold layer are visible. The patterns of these cracks are of interest for the present work as there are variations in those patterns that depend on the surface functionalization. On the other hand, the SEM micrograph taken with the secondary electron detector (SE) located at the side of the electron chamber (Figure 7b) has a significant topological contrast that contributes to the understanding of the tridimensional structure of the membrane. However, the resolution is lower than the SEM image recorded with the InLens detector (Figure 7a), therefore the informative cracks in the gold layer are not visible. Given this limiting consideration for the micrographs of the SE detector, the membrane surface was further investigated at higher magnification with the InLens detector (Figure 8).

**Figure 9:**
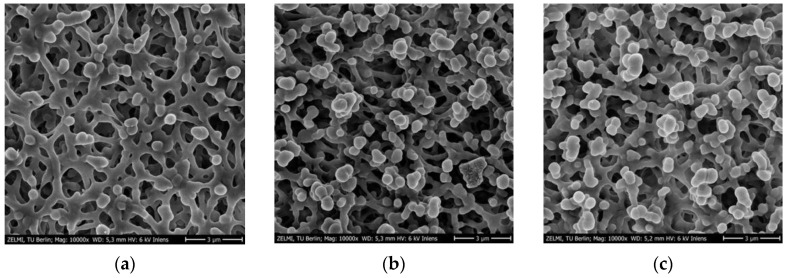
SEM micrographs at 10,000× magnification of (**a**) bare PES membrane, (**b**) chitosan functionalized PES membrane before nanoMIP immobilization, and (**c**) chitosan functionalized PES membrane after nanoMIP immobilization.

Figure 8 and Figure 9 show SEM micrographs after major membrane preparation steps at high (100,000×) and low magnifications (10,000×). Morphology changes in the sputtered gold layer were noticed in the PES membranes after the treatment steps. Particularly, the major changes were associated with the nanoMIP immobilization. The edges became sharper and the cracks in the gold layer were bigger (Figure 8c). 

Even under lower magnification, there are slight differences highlighting the blob-like structures after chitosan functionalization (Figure 9b) in comparison with the bare PES membrane (Figure 9a).

### 3.4. Filtration Experiments

#### 3.4.1. Permeation Flux 

Filtration experiments were conducted to evaluate the performance of the membranes. Filtration time and the permeate volume were recorded for every experiment to calculate the permeation flux by using Equation (2), where *J* is the permeation flux [L m^−2^ h^−1^], *V_f_* is the permeate volume [L], *A* is the active area [m^2^] and *t* is the operation time [h] [26]. The active area of the membranes was 0.0019625 m^2^.

(2) $$J=\frac{V_{f}}{A\times t}$$

Table 3 shows the permeation fluxes corresponding to different volumes of the loading solution at distinct major steps of membrane preparation. Interestingly, the permeation flux increases after chitosan functionalization. This parameter is directly related to the membrane performance in terms of volume filtered per unit time. In practical terms, a membrane would have superior performance for large-scale applications, if it is capable of filtering a larger volume of liquid (e.g., water) in the same time frame. For example, considering the measurements after filtering 10 mL loading solution, theoretically a square meter chitosan functionalized bioselective PES membrane would filter 157.64 L more than a bare PES membrane in one hour. 

According to the literature, the permeation flux increases as the hydrophilicity improves [27]. The chitosan functionalization favored the membrane performance as the permeation flux and percentage of volume recovered after filtration increased, even when the bare PES membrane was already hydrophilic. Two distinct filtration volumes were investigated: 5 mL and 10 mL. The consistent increase in the permeation flux associated with the largest loading solution volume (10 mL) after subsequent functionalization steps demonstrates a promising potential for investigating larger volumes from the milliliter to liter scale and ultimately, scaling up the membrane filter.

#### 3.4.2. Membrane Fouling Studies

Previous research has proved that hydrophilicity prevents fouling behavior [28]. To further investigate the fouling behavior of the chitosan functionalized membranes, a similar volume of the loading solution was filtered three times through the same membrane. The filtration setup shown in Figure 2 was used and the largest volume of the previous filtration experiments (10 mL) discussed in Section 3.4.1 was selected for the membrane fouling studies. In addition, control experiments were performed with a bare polymer membrane to compare the decline in the permeation flux due to fouling.

The filtration time, active area, and permeate volume were recorded for every filtration to then calculate the permeation flux. According to the literature, the permeation flux is expected to decrease as the natural organic matter (NOM) contaminants accumulate in the surface. This decrease implies that after every filtration, a lesser volume of water can be filtered for the same operation time. Therefore, the service life of the membrane shortens, highlighting that membrane fouling is one of the costliest problems in the membrane industry [17].

The flux decline ratio (FDR) is an important parameter to investigate the fouling behavior of a membrane in terms of the permeation flux reduction in comparison to a previous filtration in the same membrane. The FDR is described mathematically in Equation (3), where *J_o_* is the initial permeation flux (from the previous filtration) and *J_f_* is the final permeation flux [17].

(3) $$\mathrm{FDR}=\left(1-\frac{J_{f}}{J_{0}}\right)\times 100\%$$

Table 4 summarizes the results of the fouling behavior investigations for PES membranes. The largest decline in the permeation flux was observed in the bare polymer membranes. Therefore, the untreated membranes are more prone to fouling, thus their efficiency reduces in shorter periods of time. 

In contrast, the chitosan functionalized membranes had a smaller decrease in ratio in the permeation flux. Hence, the membrane surface accumulates fewer solids that hinder the permeation flux than the bare polymer membrane. Thus, the potential service-life of the chitosan functionalized membranes would be longer. For example, after the third filtration, the permeation flux of the functionalized PVDF membranes declined by less than 15%. 

#### 3.4.3. Filtration Studies and Uptake Capacity

Different volumes of loading solution with a known adenovirus concentration (10^5^ pfu mL^−1^) were filtered through the investigated membranes as described in Section 2.5. The permeate was collected after filtration to then quantify the adenovirus concentration left in the permeate. 

There are different methods for virus quantification by using distinct detection devices. Every device generates a particular response that is proportional to the concentration of virus. For instance, QCM measures the mass change per unit area in terms of variations in the frequency of a quartz crystal resonator [29]. 

**Figure 10:**
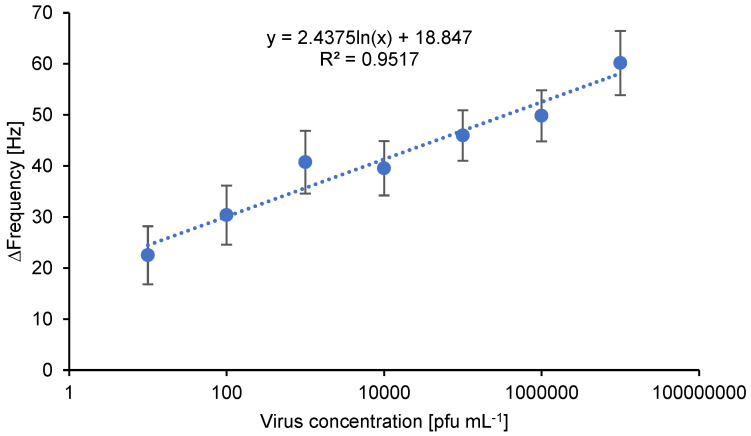
Calibration curve of human inactivated adenovirus serotype 5 from quartz crystal microbalance (QCM) measurements. Limit of detection (LOD): 10 pfu mL^−1^.

The mathematical proportionality between a detection response and the corresponding concentration is given by a calibration curve. Ideally, the detection response is linearly proportional to the concentration. Firstly, a series of adenovirus solutions are prepared by serial dilution with known concentrations that consistently increase within the range of interest for the investigations (10 to 10^7^ pfu mL^−1^). Then, the sample virus solutions are individually measured, followed by plotting the frequency change associated with each concentration (Y axis) against the known concentration for the sample (X axis) as displayed in Figure 10. 

Thereafter, the slope value (*m*) of the linear function equation (Equation (4)) is calculated by linear regression.

(4) y=mx+b 

Finally, the concentration of samples with unknown concentrations can be calculated with the frequency change from the measurement and the *m* value calculated from the calibration curve. 

The bare PES membrane performs quite well to remove low concentrations of viruses at low volumes as shown in Table 5. However, the uptake capacity decreases from 99.99% to approximately 20% when increasing the filtration volume by double (from 5 mL to 10 mL). Therefore, 79.04% of the viruses in the loading solution went through the membrane and remained in the permeate. The increase in filtration volume by a factor of two (from 5 mL to 10 mL loading solution) resulted in a significant decrease of almost 80% in the uptake capacity. This result demonstrates a major limitation for prospective upscaling of the untreated membrane, where the volume is expected to increase by a factor of ten in early stages and even hundreds in later stages.

On the other hand, the chitosan + nanoMIP functionalized PES membrane had a consistent uptake capacity. Therefore, the bioselective membrane with chitosan + nanoMIP functionalization rejected 99.99% of the viruses, even when increasing the volume by double the amount. These findings are especially favorable when considering future upscaling to filter larger volumes of a loading solution.

**Figure 11:**
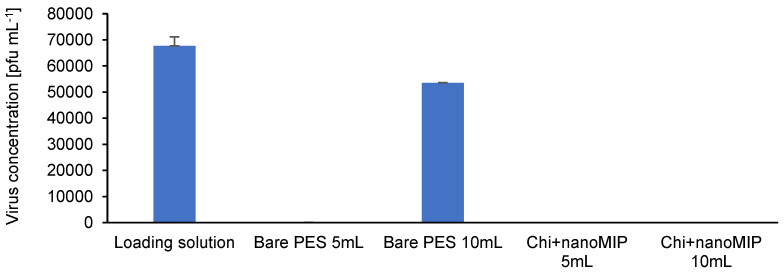
Virus concentration of the loading solution, permeates of bare PES membranes and permeates of bioselective chitosan + nanoMIP functionalized PES membranes.

The virus concentrations of the loading solution, and permeates from the bare PES membranes and bioselective chitosan + nanoMIP functionalized membranes are displayed in Figure 11. 

The significant increase in virus concentration after filtering a larger loading concentration volume through the bare PES membranes as well as the consistently low concentrations of the permeates after filtering the same loading solution through the developed bioselective membrane is similarly noticeable in Figure 11. 

Finally, the regeneration capabilities of the bioselective membrane were evaluated according to the previously reported study [30]. In general, the regeneration solution (HCl or ACN) was filtered through the membrane and then the PBS buffer was filtered. Thereafter, the virus concentration of the PBS permeate was quantified by QCM measurements to calculate the virus recovery after regeneration. The results of these investigations are displayed in Table 6.

The low recovery percentages might be associated with a high affinity between the viruses in the loading solution and the nanoMIPs immobilized on the membrane. This means that the bonds between the adenovirus and the nanoMIP-specific cavity are stronger than the interactions between the virus and the regeneration system, therefore the virus–nanoMIP bond was not easily broken by HCl or ACN. This high affinity between the virus and the nanoMIP hinders the regeneration capabilities of the membranes [21]. 

## 4. Conclusions

Novel bioselective PES membranes based on chitosan functionalization and virus-specific nanoMIP immobilization were successfully prepared. From the contact angle measurements and swelling behavior studies, it was confirmed that the membrane samples became more hydrophilic after chitosan modification. The prepared membranes were further characterized with AFM and SEM in terms of surface topography, phase distribution and surface morphology. Additionally, the RMS roughness consistently increased after every major membrane preparation step. Moreover, membrane performance was investigated in terms of permeation flux, membrane fouling, and virus quantification. The permeation fluxes increased after chitosan functionalization and the membranes were less prone to surface fouling when functionalized with chitosan. Finally, it could be demonstrated that the effective combination of chitosan hydrophilic surface modification and virus-specific nanoMIP receptor immobilization on a PES membrane enabled the successful removal of adenovirus from water up to 99.99%. Therefore, the bioselective PES membrane developed in this study provides a fundamental basis for future investigations in terms of virus selectivity and specificity, membrane regeneration, and large-scale pilot tests. It is expected that with further optimization studies, the established membrane preparation method may pave the way for significant regeneration profiles coupled with high selectivity and specificity. Moreover, the developed immobilization strategy holds great promise not only for the modification of PES membranes but also for other polymeric membranes to selectively remove viruses from contaminated water sources. 

## Figures and Tables

**Table 1:** Swelling degrees of PES membranes at main steps of membrane preparation.

Sample	W_dry_ [g]	W_wet_ [g]	Degree of Swelling
Bare PES membrane	52.5	109.7	1.08
Chitosan functionalized membrane	54.1	115.6	1.13
Chitosan + nanoMIP functionalized membrane	55.3	125.3	1.26

**Table 2:** RMS values after major functionalization steps.

Size [µm]	Bare PES Membrane		Chitosan Functionalized Membrane		Chitosan + nanoMIP Functionalized Membrane	
	Average RMS [nm]	Standard Deviation [nm]	Average RMS [nm]	Standard Deviation [nm]	Average RMS [nm]	Standard Deviation [nm]
50 × 50	391.89	24.29	416.58	24.31	448.67	26.65
10 × 10	300.58	18.09	351.46	20.21	402.87	19.36
3 × 3	188.68	12.49	221.26	9.40	229.55	21.26

**Table 3:** Permeation flux data.

Sample	V_loading solution_ [mL]	Permeation Flux [L m^−2^ h^−1^]	%Volume Recovered from Loading Solution
Bare PES membrane	5	730.76	80.00
	10	991.63	87.00
Chitosan functionalized membrane	5	1225.19	96.00
	10	1090.82	94.00
Chitosan + nanoMIP functionalized membrane	5	1118.06	86.00
	10	1149.27	88.00

**Table 4:** Fouling behavior of PES membrane samples.

Sample	Filtration Order	V_loading solution_ [mL]	Permeation Flux [L m^−2^ h^−1^]	FDR [%]
Bare PES membrane	First	10	1490.97	-
	Second	10	1031.52	30.81
	Third	10	848.49	17.74
Chitosan functionalized PES membrane	First	10	1225.18	-
	Second	10	966.14	21.14
	Third	10	842.38	12.80

**Table 5:** Membrane filtration calculations for PES membranes.

Sample	Percentage of Loading Concentration	Uptake Capacity [%]	NanoMIP Distribution Density [µg cm^−2^]
Bare PES membrane V_loading solution_ = 5 mL	1.07 × 10^−5^	99.99	no nanoMIPs
Bare PES membrane V_loading solution_ = 10 mL	79.04	20.95	no nanoMIPs
Chitosan + nanoMIP functionalized membrane V_loading solution_ = 5 mL	2.05 × 10^−8^	99.99	5.1
Chitosan + nanoMIP functionalized membrane V_loading solution_ = 10 mL	0.008	99.99	5.1

**Table 6:** Membrane regeneration studies.

Sample	Virus Recovery in Buffer after Regeneration [%]
First filtration after regeneration with HCl	0.003
Second filtration after regeneration with HCl	0.0003
First filtration after regeneration with cold ACN	2.12 × 10^−5^
First filtration after regeneration with hot ACN	1.36 × 10^−5^

## Data Availability

The data presented in this study are available on request from the corresponding author.
